# The Phenomenon of Antimicrobial Resistance in Southern Italy: An Overview of the Current Situation

**DOI:** 10.34172/apb.025.45467

**Published:** 2025-10-11

**Authors:** Stefano Ruga, Raffaele Petti, Mara Masullo, Fabio Castagna, Roberto Bava, Michelangelo Armenise, Elisabetta Labbate, Carmen Lombardi, Antonio Giordano, Massimiliano Quintiliani, Luigi Alfano, Emilia Langella, Giovanna Liguori, Renato Lombardi

**Affiliations:** ^1^Department of Health Sciences, University of Catanzaro Magna Græcia, 88100 Catanzaro, Italy; ^2^Local Health Authority, ASL, 71121 Foggia, Italy; ^3^University of Bari Aldo Moro, 70100, Bari, Italy; ^4^University Cattolica of Rome, 00168, Roma, Italy; ^5^Sbarro Institute for Cancer Research and Molecular Medicine, Center for Biotechnology, Department of Biology, College of Science and Technology, Temple University, Philadelphia, U.S.A.; ^6^Sbarro Health Research Organization ETS, 10060, Candiolo (TO), Italy; ^7^Department of Breast and Thoracic Oncology, Istituto Nazionale Tumori, IRCSS-G Pascale, 80131, Napoli, Italy; ^8^Department of Agricultural, Forestry, Food and Environmental Sciences, Università degli Studi della Basilicata Campus di Macchia Romana Via dell’Ateneo Lucano, 10 - 85100 Potenza, Italy; ^9^Local Health Authority of Foggia, Via Monte Grappa 25, 71121, Foggia, Italy

**Keywords:** Antimicrobial resistance, Demographics, Public health, Southern Italy

## Abstract

**Purpose:**

Antimicrobial resistance (AMR) is an escalating global health challenge with region-specific implications. This study investigated AMR prevalence in Southern Italy, with particular attention to demographic variables such as gender and age.

**Methods:**

A retrospective analysis of antibiograms from 146 patients (68 males and 78 females, aged 36–101 years) collected between 2022 and 2023 was conducted. Given the retrospective design and reliance on routinely collected clinical data from a local hospital microbiology laboratory, molecular analyses were not feasible, as isolates were processed solely for diagnostic purposes and not preserved.

**Results:**

The most frequently isolated pathogens were *Escherichia coli* (52.3%), *Klebsiella pneumoniae* (14.9%), *Enterococcus faecalis* (6.9%), *Proteus mirabilis* (6.3%), *Staphylococcus aureus* (4.6%), and *Pseudomonas aeruginosa* (4.3%). In males, the highest resistance rates were recorded for ciprofloxacin (47.9%), levofloxacin (47.2%), and trimethoprim/sulfamethoxazole (40.5%). Female patients showed greater resistance to amoxicillin/clavulanate, levofloxacin, and trimethoprim/sulfamethoxazole, with women≥70 years displaying particularly elevated resistance compared with age-matched men and younger females.

**Conclusion:**

Despite the absence of molecular data, phenotypic surveillance through antibiograms remains a critical tool for monitoring AMR trends in underrepresented regions. Incorporating gender-specific differences into clinical practice may improve therapeutic efficacy and stewardship strategies. These findings provide a foundation for future molecular and epidemiological investigations.

## Introduction

 Antibiotics represent one of the most essential classes of therapeutic agents for the treatment of a broad spectrum of infections and have played a pivotal role in safeguarding and improving human health. However, in recent years, their inappropriate use and overuse have contributed substantially to the emergence and spread of antimicrobial resistance (AMR).^1,2^ AMR arises when microorganisms—including bacteria, fungi, and parasites—acquire the capacity to withstand and continue proliferating despite exposure to antimicrobial agents that were once effective against them. AMR represents an increasingly critical threat to modern medicine, as it undermines the efficacy of standard therapeutic regimens and compromises the success of even routine medical procedures, thereby contributing to the persistence of infections that are difficult to treat and control.^3-5^ Current estimates suggest that approximately 700,000 deaths worldwide each year are attributable to antibiotic ineffectiveness, a number projected to rise almost fifteen-fold over the next 25 years if current trends persist..^6,7^

 The World Health Organization (WHO) has identified AMR as one of the most serious threats to global health. In response, it has developed a Global Action Plan designed to increase awareness through education and information initiatives, while simultaneously promoting the prudent and optimized use of antimicrobials across both human and veterinary medicine.^8^

 The consequences of AMR include heightened morbidity and mortality, substantial increases in healthcare expenditures, and the widening of existing disparities in access to medical care.^9^

 The emergence and spread of AMR can be attributed to multiple interrelated factors, including the inappropriate and excessive use of antibiotics in human medicine, inadequate hygiene and infection prevention measures, and the widespread overuse of antimicrobials in animal husbandry and agriculture.^10^ Notably, numerous studies have demonstrated a strong association between elevated antibiotic consumption and the emergence of AMR. Countries with high levels of antibiotic use report substantially greater rates of therapeutic failure, accompanied by marked increases in infection incidence and mortality, with estimates reaching as high as 30,000 deaths annually.^11,12^

 In response, several European countries have introduced more stringent regulatory protocols to limit antibiotic consumption, resulting in demonstrable reductions.^13,14^

 The global dissemination of antibiotic-resistant bacteria is further exacerbated by intensified patterns of human migration. This phenomenon poses particular challenges for countries that have implemented robust antibiotic stewardship programs. Migrants and travelers may serve as carriers, introducing resistant bacterial strains that were previously absent in the host country, thereby undermining public health initiatives designed to mitigate antibiotic misuse and aggravating the AMR crisis. Moreover, migration contributes to greater bacterial heterogeneity, complicating surveillance and containment efforts. Addressing this multifaceted challenge necessitates the development of coordinated international strategies grounded in an integrated, multidisciplinary framework.^15,16^

 An often-neglected dimension of AMR concerns its intersection with gender, a research domain that remains insufficiently examined. The existing scientific literature offers limited evidence on potential associations between gender and AMR, despite their considerable implications for public health. Notably, the majority of epidemiological investigations addressing AMR at a global scale fail to provide gender-disaggregated data, thereby constraining a nuanced understanding of this relationship.^3,17^ The systematic collection and analysis of sex-disaggregated data hold significant potential for informing the development of more targeted and effective strategies for the prevention and control of AMR. Advancing research in this domain is therefore essential to address existing knowledge gaps and to promote a more equitable and evidence-based approach. As emphasized by the WHO, antibiograms—which offer detailed insights into patterns of bacterial resistance—constitute a critical tool for optimizing clinical decision-making and assessing the effectiveness of intervention measures.^18^

 Southern Italy presents a set of distinctive socio-economic and environmental conditions that are likely to influence local patterns of AMR. The paucity of region-specific studies underscores the need for comprehensive research to elucidate both the extent and the determinants of AMR within this population. The primary aim of the present study was to investigate the prevalence and characteristics of AMR, with particular attention to demographic variables such as gender and age. These factors are essential for identifying population subgroups at greater risk and for designing targeted interventions aimed at mitigating the spread of resistance. In light of the increasing global burden of AMR, understanding its dynamics within the Southern Italian context is critical for the development of more effective strategies for infection prevention and treatment, ultimately strengthening public health at both regional and national levels.

 To establish a coherent theoretical foundation, this study draws upon two complementary frameworks. First, AMR is conceptualized as a multifactorial phenomenon requiring a comprehensive perspective such as the *One Health* approach, which emphasizes the intrinsic interconnections between human, animal, and environmental health. Misuse of antibiotics across these domains accelerates the emergence and dissemination of resistance, thereby requiring coordinated, multisectoral responses.^19^ Second, the role of social determinants of health—including disparities in access to healthcare, sanitation, and education—is recognized as shaping both exposure risks and treatment outcomes. Gender dynamics add further complexity; emerging literature highlights how sociocultural norms, behavioral patterns, and systemic discrimination contribute to divergent antibiotic use and resistance profiles between men and women.^20^ Incorporating these conceptual models strengthens the rationale for focusing on demographic variables such as gender and age in the present study.

 Despite the critical global challenge posed by antimicrobial resistance (AMR), there is limited evidence regarding how demographic factors, particularly gender and age, influence the prevalence and patterns of resistance at the local level. This knowledge gap is especially pronounced in underrepresented regions, where comprehensive molecular analyses are often not feasible due to logistical and infrastructural constraints. In this context, the present study leverages routinely collected clinical data from a local hospital microbiology laboratory in Southern Italy, using phenotypic surveillance through antibiograms as a valuable tool to assess resistance trends. Given the retrospective nature of the study and the absence of stored bacterial isolates for molecular investigation, the research focuses on characterizing the distribution and frequency of AMR across outpatient populations while examining potential associations with gender and age. By doing so, the study aims to provide foundational epidemiological insights that can inform clinical decision-making and antimicrobial stewardship efforts, while laying the groundwork for more detailed molecular investigations in future research.

## Materials and Methods

###  Study design and sample selection

 Patients’ culture data were obtained from records maintained by the Local Health Authority of the Province of Foggia (Puglia, Italy). Biological samples were processed at the Clinical Analysis Laboratory and Clinical Pathology Center of the Presidio Ospedaliero ‘San Camillo De Lellis’ in Manfredonia (Foggia). The study included samples collected from microbiological examination requests for 54 outpatients, whereas specimens from patients admitted to the hospital’s individual Operating Units were excluded.

 The analyzed samples comprised various specimen types, predominantly urine cultures (98.3%), with the remainder consisting of swabs from skin, sputum, and pharyngeal sites. Samples were obtained from both male and female patients, totaling 146 individuals (68 males and 78 females). A total of 350 specimens were examined, including 163 from male patients and 187 from female patients, collected between 2022 and 2023. Patient ages ranged from 36 to 101 years, with a mean age of 74 years. Of the total cohort, 48 patients were aged 70 years or younger (20 males, 28 females), while 98 patients were aged 70 years or older (48 males, 50 females).

 Through Edotto’s Directional System (DISAR) and an evaluation of records from general practitioners (GPs) in DSS 54, the six most prescribed antibiotics during the reporting period were identified: AMC, CIP, FO, LEV, TMP/SMX and CLA.

###  Sample analysis

 For each specimen, biochemical characterization was performed by a trained bacteriologist. The procedure included sequential steps of seeding, growth, isolation, enumeration, and direct or presumptive pathogen identification using CPS Elite solid chromogenic agar media. Subsequently, antibiotic susceptibility testing (AST) was conducted utilizing the semiautomatic MicroScan AutoSCAN-4 system (Beckman Coulter, USA) in conjunction with the microdilution method.

 The methodology commenced with the preparation of a standardized bacterial inoculum. One to two well-isolated colonies from a fresh culture were collected using a PROMPT sterile needle, suspended in sterile saline, and homogenized to achieve a uniform suspension. The resulting suspension was then applied to 96-well microplate panels, designed according to European Committee on Antimicrobial Susceptibility Testing (EUCAST) standards. Each well contained lyophilized antibiotics at varying concentrations and additional growth substrates. Control wells included a negative control and a growth control, with turbidity in the latter confirming microbial viability and replicative capacity.

 Rehydration of the wells was performed by dispensing approximately 120–125 µL of the prepared suspension using a RENOK device, followed by incubation at 37°C for 24 hours. Panels were subsequently read using the AutoSCAN-4 system, an automated photometric reader capable of detecting bacterial growth and colorimetric changes in biochemical reactions. The optical system comprises a tungsten lamp, six interference filters (405–620 nm), and 96 optical fibers, allowing simultaneous measurement of all wells via photodiodes. Turbidimetric readings were also obtained using a 590 nm interferential filter.

 The system provided identification percentages within seconds, which were then interpreted against EUCAST-defined clinical breakpoints to classify microorganisms as susceptible, intermediate, or resistant. Data interpretation, reporting, and archiving were managed using LabPro^TM^ software.

###  Statistical analyses

 Data were collected in Microsoft (MS) Excel and analyzed with Statistical Package for the Social Sciences (SPSS) version 27 software (IBM Corp., Armonk, NY, USA). The chi-square test (χ2) was used to compare differences in susceptibility among patients of different genders and ages. A multivariate logistic regression analysis was also performed to assess the independent effect of age and gender on susceptibility within the sample. Values were considered significant when *P* < 0.05.

## Results

 A total of 146 patients were enrolled in the study, consisting of 68 males (46.6%) and 78 females (53.4%). Between 2022 and 2023, 350 clinical samples were collected, of which 163 (46.6%) originated from male patients and 187 (53.4%) from female patients. The vast majority of specimens were urine samples (98.3%), while the remaining samples were derived from skin swabs, sputum, or pharyngeal swabs. The age of participants ranged from 36 to 101 years, with a mean age of 74 years. Of the total cohort, 32.9% were between 36 and 70 years of age (20 males and 28 females), whereas 67.1% were between 71 and 101 years of age (48 males and 50 females) (Table 1).

**Table 1:** Demographic characteristics of patients.

**Age**	**Male (N)**	**Male (%)**	**Female (N)**	**Female (%)**	**Total**
36-70	20	41.7	28	58.3	48
71-101	48	49	50	51	98

 Antibiogram analysis indicated that the most prevalent bacterial isolates were *Escherichia coli* (52.29%), followed by *Klebsiella pneumoniae* (14.86%), *Enterococcus faecalis* (6.86%), *Proteus mirabilis* (6.29%), *Staphylococcus aureus* (4.57%), and *Pseudomonas aeruginosa* (4.29%). No statistically significant differences in bacterial distribution were observed according to gender or age (Figure S1, Table S1 – Supplementary File 1).

**Figure 1:**
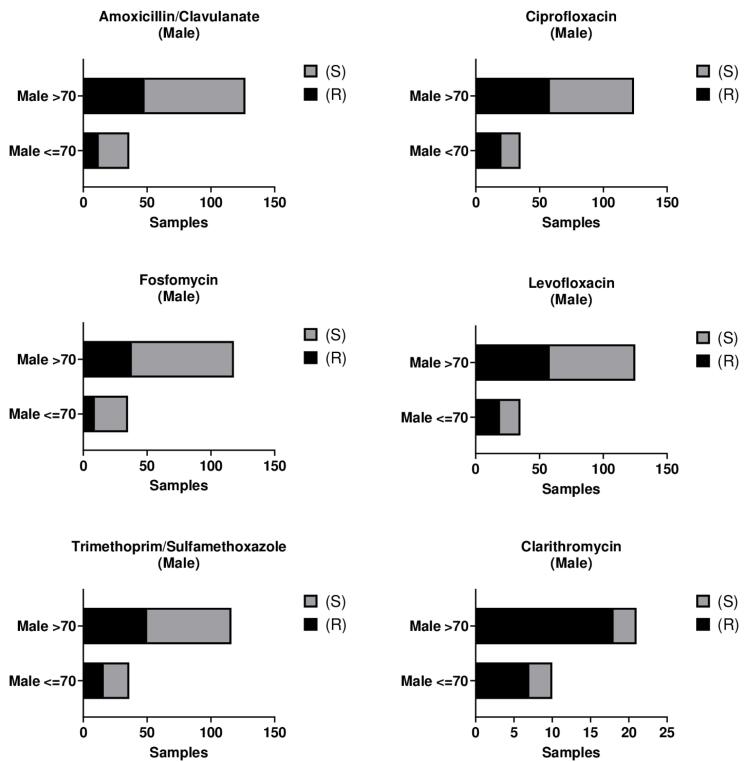
Comparison of antibiotic susceptibility in male patients according to age. S = susceptibility, R = resistance

 Among male patients, the bacterial isolates demonstrated the highest resistance to ciprofloxacin (CIP) (47.9%), followed by levofloxacin (LEV) (47.2%), trimethoprim/sulfamethoxazole (TMP/SMX) (40.5%), and amoxicillin/clavulanate (AMC) (36.8%). The lowest resistance rates were observed for fosfomycin (FO) (28.8%) and clarithromycin (CLA) (6.1%) (Figure S2, Table S2 – Supplementary File 1). No statistically significant differences in resistance profiles were detected between male patients aged ≤ 70 years (36–70 years) and those aged > 70 years (Figure 1, Table S2; Supplementary File 1).

 In female patients, bacterial isolates exhibited the highest levels of resistance to CIP (61.0%) and LEV (59.4%). Resistance to TMP/SMX and AMC was also substantial, at 55.6% and 53.5%, respectively. The lowest resistance rates were observed for FO (25.7%) CLA (13.4%) (Table 2; Figure S3, Table S3 – Supplementary File 1).

**Table 2:** Antibiotic susceptibility in female patients.

**Antimicrobial**	**Variable**	**OR**	**95% CI**	***P*** ** value**
Amoxicillin/Clavulanate	Age ( < 70)	1.006	0.819 - 1.183	0.941
	Age ( > 70)	1.282	1.021 - 2.197	0.143
Ciprofloxacin	Age ( < 70)	1.025	0.874 - 1.201	0.754
	Age ( > 70)	1.507	0.976 - 2.635	0.091
Fosfomycin	Age ( < 70)	1.060	0.923 - 1.248	0.421
	Age ( > 70)	1.058	0.776 - 1.670	0.734
Levofloxacin	Age ( < 70)	1.089	0.974 - 1.254	0.175
	Age ( > 70)	1.106	0.957 - 1.316	0.197
Trimethoprim/Sulfamethoxazole	Age ( < 70)	1.292	1.039 - 1.779	0.054
	Age ( > 70)	2.372	1.216 - 6.547	0.041*
Clarithromycin	Age ( < 70)	n.e.	n.e.	n.e.
	Age ( > 70)	n.e.	n.e.	n.e.

n.e. = not estimable due to insufficient sample size for logistic regression. *P < 0.05. Multivariate logistic regression test.

**Figure 2:**
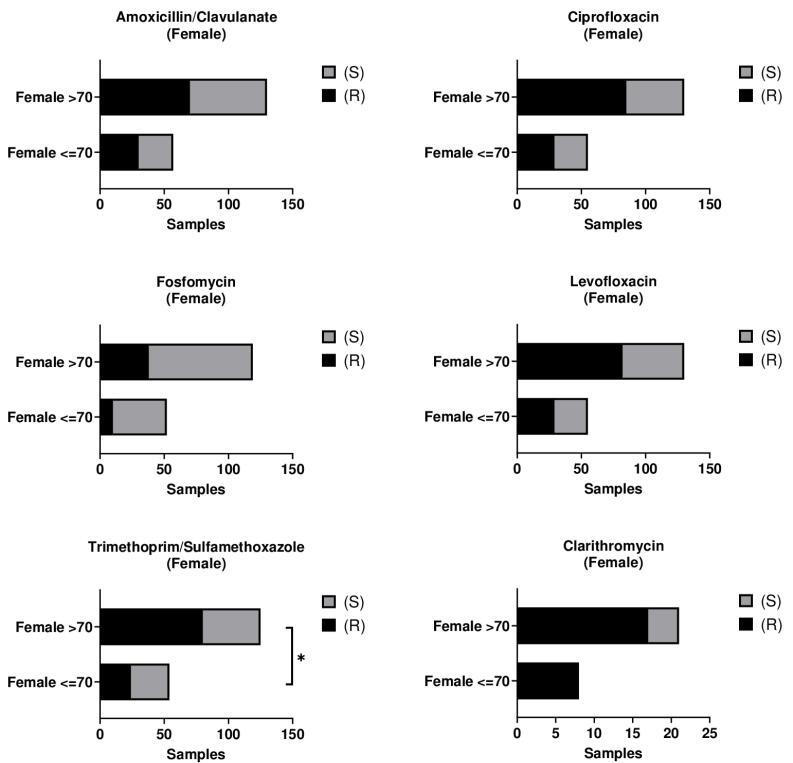
Comparison of antibiotic susceptibility in female patients according to age. S = susceptibility, R = resistance. *P < 0.05

 Age-stratified analysis demonstrated a significantly higher resistance to TMP/SMX among female patients aged > 70 years compared with those aged ≤ 70 years (*P* < 0.05). For all other antibiotics, no significant differences were observed between the two age groups (Figure 2; Table S3 – Supplementary File 1).

 To identify independent predictors of antimicrobial resistance, a multivariate logistic regression analysis was performed. The results confirmed that the significant associations identified by the chi-square test (Table S3 – Supplementary File 1) remained robust after adjustment for potential confounders. Specifically, resistance to TMP/SMX was significantly higher in female patients over 70 years of age compared to their younger counterparts (*P* = 0.041). Furthermore, female gender emerged as an independent predictor of resistance to AMC, CIP, and LEV. Detailed regression outputs, including odds ratios (ORs), 95% confidence intervals (CIs), and *P* values for each predictor, are provided in Tables 2-4.

**Table 3:** Antibiotic susceptibility and gender difference

**Antimicrobial**	**Variable**	**OR**	**95% CI**	***P *** **value**
Amoxicillin/Clavulanate	Gender	2.196	1.220 - 4.040	0.009*
	Age	1.033	0.992 - 1.076	0.113
Ciprofloxacin	Gender	2.112	1.093 - 4.180	0.028*
	Age	1.023	0.995 - 1.052	0.101
Fosfomycin	Gender	1.002	0.607 - 1.650	0.993
	Age	1.001	0.978 - 1.023	0.938
Levofloxacin	Gender	1.866	1.120 - 3.161	0.018*
	Age	1.003	0.982 - 1.025	0.777
Trimethoprim/Sulfamethoxazole	Gender	2.138	1.216 - 3.812	0.009*
	Age	1.012	0.990 - 1.035	0.266
Clarithromycin	Gender	1.002	0.123 - 5.944	0.998
	Age	1.013	0.963 - 1.073	0.619

*P < 0.05. Multivariate logistic regression test.

**Table 4:** Antibiotic susceptibility and gender difference in patients aged > 70 years

**Antimicrobial**	**Variable**	**OR**	**95% CI**	***P*** ** value**
Amoxicillin/Clavulanate	Gender	2.736	1.368 - 5.608	0.005**
	Age	1.011	0.956 - 1.070	0.707
Ciprofloxacin	Gender	2.637	1.370 - 5.155	0.004**
	Age	1.056	0.996 - 1.121	0.067
Fosfomycin	Gender	1.049	0.572 - 1.930	0.876
	Age	1.016	0.963 - 1.073	0.555
Levofloxacin	Gender	2.433	1.309 - 4.580	0.005**
	Age	1.024	0.973 - 1.078	0.365
Trimethoprim/Sulfamethoxazole	Gender	3.000	1.554 - 5.910	0.001**
	Age	1.013	0.959 - 1.069	0.641
Clarithromycin	Gender	1.077	0.124 - 7.609	0.940
	Age	1.008	0.877 - 1.165	0.910

**P < 0.01. Multivariate logistic regression test.

 A pooled analysis of all samples from both sexes revealed the highest resistance rates for CIP (53.9%) and LEV (53.7%). Resistance to TMP/SMX and AMC was also substantial, at 48.6% and 45.7%, respectively. The lowest resistance rates were observed for FO (27.1%) and CLA (14.3%) (Figure S4, Table S4 – Supplementary File 1).

**Figure 3:**
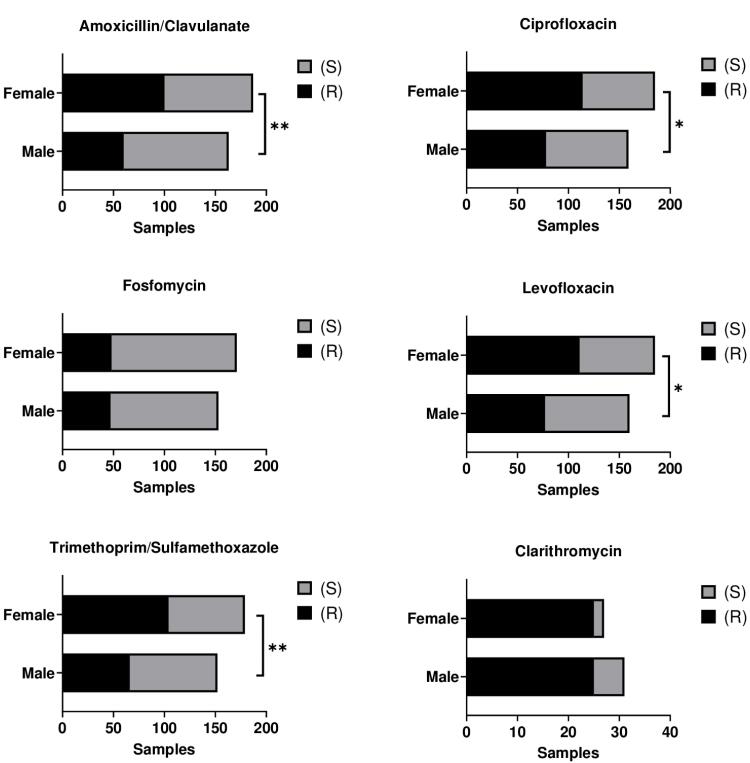
Comparison of antibiotic susceptibility between male and female patients. S = susceptibility, R = resistance. *P < 0.05, **P < 0.01

 Comparative analysis between sexes demonstrated significantly higher resistance among female patients than male patients for AMC and TMP/SMX (*P* < 0.01). Resistance to CIP and LEV was also significantly higher in female subjects (*P*< 0.05). No statistically significant sex-related differences were detected for FO or CLA (Figure 3). These findings, initially identified using the chi-square test, were subsequently confirmed through multivariate logistic regression analysis (Table 3).

 Among patients aged ≤ 70 years (36–70 years), CIP exhibited the highest resistance rate at 52.7%, followed by LEV at 51.6%, AMC at 45.1%, and trimethoprim/sulfamethoxazole (TMP/SMX) at 43.0%. The lowest resistance rates were observed for FO and CLA, at 20.4% and 16.1%, respectively (Figure S5 – Supplementary File 1).

**Figure 4:**
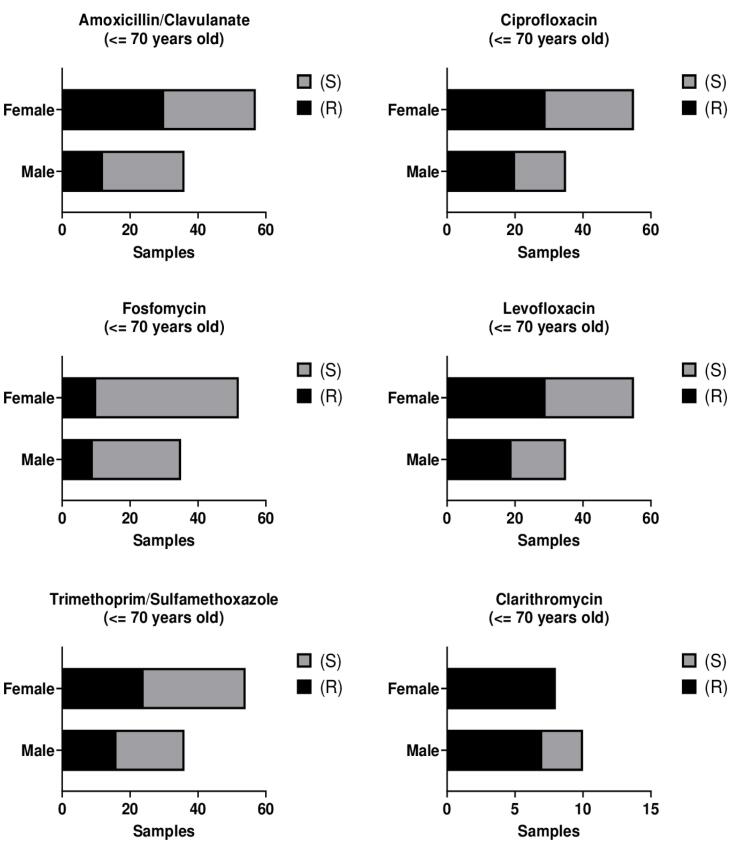
Comparison of antibiotic susceptibility in male and female patients aged ≤ 70 years old. S = susceptibility, R = resistance

 No statistically significant differences in resistance were observed between male and female patients within this age group (36–70 years) across the antibiotics analyzed (Figure 4, Table S5).

 Among patients aged > 70 years, CIP exhibited the highest resistance rate at 55.6%, followed by LEV at 54.5%, TMP/SMX at 50.6%, and AMC at 45.9%. The lowest resistance rates were observed for FO and CLA, at 29.6% and 13.6%, respectively (Figure S6 – Supplementary file 1).

**Figure 5:**
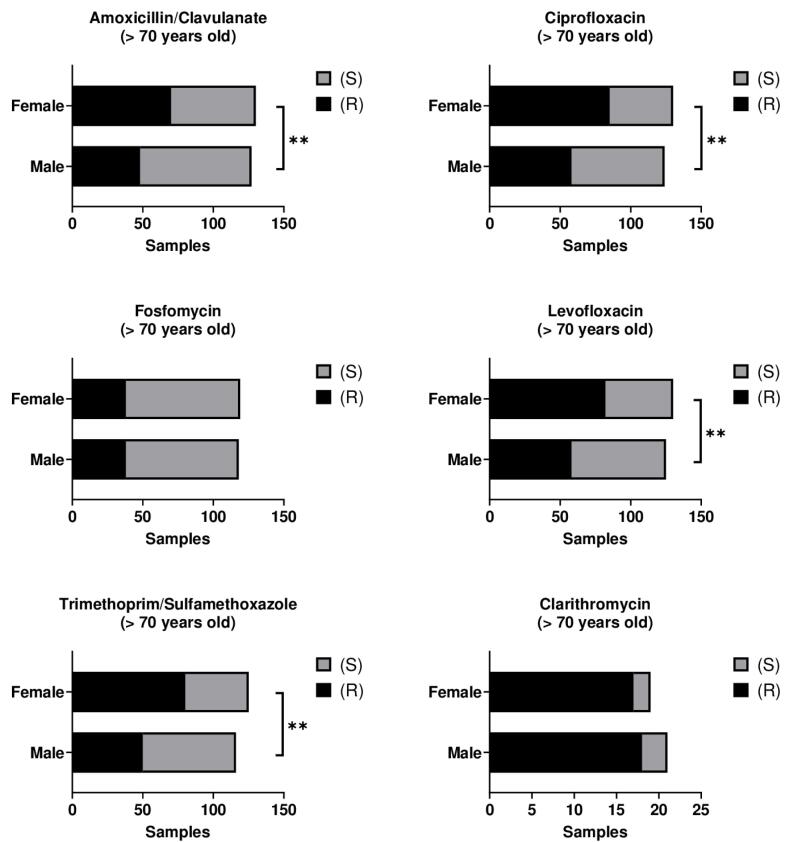
Comparison of antibiotic susceptibility in male and female patients aged > 70 years old. S = susceptibility, R = resistance. **P < 0.01

 Within this age group, resistance to AMC and CIP was significantly higher in female patients compared with their male counterparts (*P* < 0.01). Similarly, resistance to LEV and TMP/SMX was also greater in females over 70 years of age relative to males of the same age group (*P* < 0.01) (Figure 5, Table S6 – Supplementary File 1). These findings, initially identified using the chi-square test, were subsequently confirmed through multivariate logistic regression analysis (Table 4).

## Discussion

 Antibiotics are routinely prescribed for the management of diverse infectious diseases and continue to represent a cornerstone of contemporary medical practice. Nevertheless, their overuse and inappropriate administration are progressively undermining their therapeutic efficacy.^21^ In the present study, we identified notable resistance patterns among the principal bacterial pathogens associated with clinical infections. The most frequently isolated organisms included *Escherichia coli*, *Klebsiella pneumoniae*, *Enterococcus faecalis*, *Proteus mirabilis*, *Staphylococcus aureus*, and *Pseudomonas aeruginosa*, underscoring their central role in shaping the regional antimicrobial resistance profile.

 These isolates demonstrated particularly elevated resistance rates to several commonly prescribed agents, notably AMC, CIP, FO, LEV, TMP/SMX and CLA.^22^

 AMC is among the most extensively prescribed antibiotics worldwide, owing to its broad clinical applicability. This combination pairs amoxicillin, a β-lactam antibiotic of the penicillin class, with clavulanic acid, a β-lactamase inhibitor. Amoxicillin demonstrates activity against a wide range of gram-positive and gram-negative organisms and is frequently employed in the treatment of respiratory tract infections, urinary tract infections, otitis media, and sinusitis through inhibition of bacterial cell wall synthesis, ultimately leading to cell lysis and death. However, its therapeutic efficacy is often compromised by the production of β-lactamase enzymes in certain bacterial strains, which hydrolyze and inactivate the antibiotic. Clavulanic acid, although devoid of significant intrinsic antimicrobial activity, counteracts this mechanism by inhibiting β-lactamase enzymes, thereby protecting amoxicillin from enzymatic degradation and extending its antibacterial spectrum to include resistant strains. Consequently, the amoxicillin–clavulanic acid formulation remains a key therapeutic option, offering enhanced coverage and improved efficacy against β-lactamase–producing pathogens.^23^ Despite its broad therapeutic utility, the widespread and often inappropriate use of AMC has rendered it one of the antibiotics most affected by resistance. In the present study, resistance to AMC was observed in 45.7% of the tested isolates, representing one of the highest resistance rates among the agents evaluated. Moreover, AMC resistance was significantly more prevalent in female patients compared with males. This finding is consistent with existing evidence indicating that women experience a higher incidence of urinary tract infections, with the risk increasing markedly with advancing age.^24^ The elevated resistance rates observed among women, particularly those over 70 years of age, can thus be plausibly explained. A recent retrospective study conducted in 2024 on 524 female patients treated with antibiotics for urinary tract infections identified *Escherichia coli* as the most frequently isolated pathogen, a finding consistent with our results. That study further reported high resistance rates to AMC, LEV, and TMP/SMX, corroborating the resistance patterns demonstrated in our analysis.^25^

 Similarly, the study conducted by Meres et al, which investigated women over 18 years of age with urinary tract infections, reported that *Escherichia coli* and *Klebsiella* species exhibited the highest resistance rates to AMC.^26^

 Another study examining both pregnant and non-pregnant women with cystitis or bacteriuria reported that the same bacterial species exhibited higher resistance rates to AMC among non-pregnant women.^27^

 CIP, a fluoroquinolone antibiotic, is widely employed in the treatment of various bacterial infections, including urinary tract, respiratory, and bone infections. Its inclusion on the World Health Organization’s List of Essential Medicines underscores its critical clinical importance. However, extensive and prolonged use of CIP has contributed to a marked increase in bacterial resistance. This resistance not only compromises therapeutic outcomes in individual patients with severe infections but also poses broader public health challenges by restricting effective treatment options for common and otherwise manageable infections.^28^

 In the present analysis, CIP demonstrated the highest resistance rates, affecting 47.9% of isolates from male patients and 61% from female patients. Resistance was especially pronounced among women over 70 years of age, consistent with previous reports. A recent systematic review has highlighted a concerning upward trend in fluoroquinolone resistance, particularly to CIP, across Europe, Asia, and North America, with women disproportionately affected.^29^ Sanchez et al documented a progressive increase in resistance to CIP and TMP/SMX among *Escherichia coli* isolates from adult female patients between 2003 and 2012.^30^

 Similarly, in the present study, resistance to TMP/SMX was notably high in female patient samples, reaching 54.7%, with significant gender differences persisting among individuals over 70 years of age. LEV, another fluoroquinolone that inhibits bacterial DNA synthesis and induces cell death, demonstrated the second-highest resistance rate following CIP, with an overall resistance of 53.7%. Resistance among male patients was 47.2%, whereas female patients exhibited even higher rates at 59.4%. These findings are consistent with previous research conducted between 2005 and 2009, which reported substantial LEV resistance among *Escherichia coli* isolates, predominantly affecting female patients.^31^ Wang et al reported analogous findings in female patients with *Helicobacter pylori* infections, documenting elevated LEV resistance rates, particularly among women aged 40–60 years.^32^ In contrast, the present study observed the highest resistance rates among women over 70 years of age.

 FO, a broad-spectrum antibiotic commonly employed in the treatment of urinary tract infections, demonstrated the lowest resistance rate in our analysis, with an average of 27.1%. Previous research analyzing over 2,600 urine samples reported resistance rates ranging from 3% to 17%, depending on the bacterial species.^33^

 Another study underscored the high therapeutic efficacy of fosfomycin in the treatment of urinary tract infections, highlighting its preserved susceptibility among uropathogens.^25^ Nevertheless, prudent use is essential to maintain its clinical effectiveness.

 CLA, a macrolide antibiotic, is frequently employed in the treatment of respiratory and skin infections, as well as for the eradication of *Helicobacter pylori*. A 2023 study assessing *H. pylori* susceptibility to CLA reported a resistance rate of 12.1% among 141 isolates.^34^ In a study analyzing 50 bacterial isolates treated with CLA for the eradication of *Staphylococcus aureus* and *Escherichia coli*, resistance rates of 22.23% and 35.30% were observed, respectively.^35^ In the present analysis, 14.3% of isolates demonstrated resistance to CLA, consistent with a meta-analysis encompassing approximately 53,000 patients, which reported CLA resistance rates ranging from 10% to over 15%, depending on the geographic region.^36^

 The resistance patterns observed in Southern Italy in our study are consistent with those reported in other recent regional investigations. For example, an analysis of clinical *Klebsiella pneumoniae* isolates from Salerno (2015–2020) documented carbapenem resistance rates of 40–44% and extended-spectrum β-lactamase (ESBL) production in 20–22% of isolates.^37^ These findings underscore the widespread dissemination of resistant strains in the region and highlight the critical importance of ongoing surveillance.

 Additionally, a large survey of approximately 2,700 community-acquired urinary tract infection samples in Campania (2019–2020) reported a high prevalence of *Escherichia coli* (72%) and *Klebsiella pneumoniae* (12%) among the isolates.^38^ Notably, the study also identified a substantial capacity for biofilm production (~30%) among the isolated strains, suggesting a potential mechanism contributing to the phenotypic resistance patterns observed in our samples. These findings provide contextual support for our results and reinforce the hypothesis that biofilm formation may influence the development and persistence of antimicrobial resistance.

 The application of a multivariate logistic regression model was instrumental in addressing the limitations inherent in the initial univariate analysis, enabling the identification of independent predictors of antimicrobial resistance. This more rigorous analytical approach confirmed that certain demographic factors are not merely associated with resistance but represent independent risk factors. Specifically, our analysis demonstrated that female sex and advanced age ( > 70 years) are significant predictors of particular resistance profiles, even after adjustment for other potential confounders.

 These observations underscore the critical influence of demographic variables, such as age and gender, on antibiotic susceptibility. Gender-related differences may reflect multiple contributing factors, including higher rates of healthcare consultation among women.^39^ Indeed, a European meta-analysis reported that antibiotic prescription rates among women were up to 44% higher than those observed in men.^40^

 In 2018, the WHO highlighted the critical importance of addressing gender-related disparities in antibiotic resistance to inform national strategies and mitigate this global public health challenge.^41^ These observations underscore the necessity of gender-specific research to guide the development of tailored therapeutic strategies. Although molecular analyses were not conducted in the present study, hypotheses regarding the underlying mechanisms of resistance can be formulated based on recent Italian literature concerning predominant clonal lineages and associated resistance genes. For instance, in the regions of Calabria and Puglia, carbapenem-resistant *Klebsiella pneumoniae* isolates have been characterized as ST101 and ST307 clones, harboring the bla_KPC-2 and bla_KPC-3 resistance determinants.^42^ Similarly, a study conducted in the Valle d’Aosta region on carbapenem-resistant Enterobacteriaceae (CRE) reported a high prevalence of bla_VIM-1 and bla_KPC-2 genes, with evidence suggesting plasmid-mediated horizontal transfer among different strains.^43^ These findings indicate that the elevated resistance rates observed in our study may be linked to the dissemination of high-risk clonal lineages and the prevalence of specific resistance determinants, such as bla_KPC, which are widely distributed across Italy.

 This study has several important limitations that should be considered when interpreting the findings. First, the data were collected from a single hospital in Manfredonia, which may not fully represent the diversity of AMR patterns across Southern Italy, where regional variations in prescribing practices, patient demographics, and healthcare infrastructure may influence resistance trends. Second, the overwhelming predominance of urine cultures (98.3% of samples) means that the results primarily reflect resistance patterns in urinary tract infections, limiting extrapolation to other infection types such as respiratory, bloodstream, or wound infections. Third, the lack of molecular characterization—including genotypic resistance determinants or bacterial strain typing—prevents mechanistic insights into the emergence and spread of specific resistance phenotypes.

 While the study’s findings are based on data from a single hospital in Manfredonia, which may limit generalizability to broader populations, the observed resistance patterns are consistent with regional and national trends reported in other studies. Consequently, these results provide valuable insights into local antimicrobial resistance dynamics and may serve as a reference point for future multicenter investigations aimed at validating and extending these observations.

 Despite these limitations, the study provides valuable phenotypic surveillance data at a local level, offering a critical foundation for clinical decision-making and antimicrobial stewardship. To maximize the utility of these findings, several concrete actions are warranted: 1. Strengthen Local Surveillance: Systematically collect and store bacterial isolates from routine clinical samples to enable longitudinal phenotypic and future molecular analyses. This will improve the detection of emerging resistance patterns and guide empiric therapy decisions; 2. Enhance Antimicrobial Stewardship Programs (ASP): Integrate local AMR data into hospital and regional stewardship protocols to optimize antibiotic prescribing practices, particularly for urinary tract infections and other prevalent infections; 3. Targeted Education for Healthcare Professionals: Provide continuous training on the rational use of antibiotics, interpretation of antibiograms, and infection prevention practices to reduce unnecessary prescriptions and improve patient outcomes; 4. Public Awareness and Patient Education: Implement campaigns to inform patients about the risks of inappropriate antibiotic use and the importance of adherence to prescribed treatments; 5. Policy and Regulatory Support: Encourage policymakers to develop evidence-based guidelines, allocate resources for laboratory infrastructure, and support surveillance networks to facilitate timely reporting and intervention; 6. Promote Multidisciplinary Collaboration: Foster coordination among clinicians, microbiologists, epidemiologists, and public health authorities to ensure a One Health–oriented approach to AMR management; 7. Plan for Future Research: Design multicenter studies incorporating molecular characterization of isolates to elucidate resistance mechanisms and validate phenotypic surveillance data.

 By adopting these measures, hospitals and health authorities can improve antibiotic stewardship, limit the spread of resistant pathogens, and provide a framework for sustainable AMR management and public health protection.

## Conclusion

 Antimicrobials have been a transformative force in modern medicine, revolutionizing the treatment of previously fatal conditions and significantly improving survival rates, quality of life, and the safety of complex medical procedures such as surgery and cancer therapies. However, the escalating prevalence of AMR threatens to undermine these achievements, potentially returning medicine to a time when even common infections carried high mortality.

 Despite the global urgency of AMR, limited evidence exists on how demographic factors—particularly gender and age—shape the prevalence and distribution of resistance in local contexts. This gap is especially pronounced in underrepresented regions, where molecular studies are often unfeasible due to infrastructural and logistical constraints. In this setting, the present study utilized routinely collected clinical data from a hospital microbiology laboratory in Southern Italy, employing phenotypic surveillance through antibiograms to characterize resistance patterns. Although retrospective in nature and lacking stored bacterial isolates for molecular analyses, the study provides foundational epidemiological insights into AMR trends in outpatient populations, with notable differences observed across gender and age groups—particularly among women over 70 years of age.

 These findings carry direct implications for clinical and public health practice. For healthcare professionals, prudent and evidence-based prescribing is critical, guided by local antibiogram data and accompanied by patient education on adherence and responsible antibiotic use. For policymakers, targeted awareness campaigns addressing age- and gender-specific vulnerabilities, together with surveillance systems disaggregating data by demographic factors, could support more precise interventions. Antimicrobial stewardship programs must integrate these insights into region-specific protocols, update recommendations based on evolving resistance trends, and provide ongoing professional training with attention to demographic differences in resistance dynamics.

 To maximize the utility of these findings, several concrete actions are warranted. These include: strengthening local surveillance through systematic collection and storage of bacterial isolates; integrating AMR data into stewardship protocols; providing continuous training for healthcare professionals; enhancing patient education and public awareness campaigns; supporting evidence-based policy and laboratory infrastructure; fostering multidisciplinary collaboration through a One Health approach; and planning multicenter molecular studies to further elucidate resistance mechanisms.

 By adopting these measures, hospitals and health authorities can not only improve antibiotic stewardship and limit the spread of resistant pathogens but also build a sustainable framework for AMR management. Crucially, the present study underscores the importance of incorporating demographic dimensions into surveillance and intervention strategies, thereby laying the groundwork for more detailed molecular investigations and more equitable public health protection in the future.

## Competing Interests

 The authors declare no conflicts of interest.

## Data Availability Statement

 Data are available upon request to Renato Lombardi.

## Ethical Approval

 Not Applicable.

## Supplementary Files


Supplementary file 1 contains Figures S1-S6 and Tables S1-S6.
