# Supplementary material for: The Phenomenon of Antimicrobial Resistance in Southern Italy: An Overview of the Current Situation
Source: Adv Pharm Bull. 2025 Oct 11;15(4):871–82. doi: 10.34172/apb.025.45467 (PMC12980278; doi:10.34172/apb.025.45467)
Supplement: Supplementary file 1 — contains Figures S1-S6 and Tables S1-S6. [file apb-15-871-s001.pdf]

Adv Pharm Bull. 2025;15(4):871-S1
doi: 10.34172/apb.025.45467
https://apb.tbzmed.ac.ir

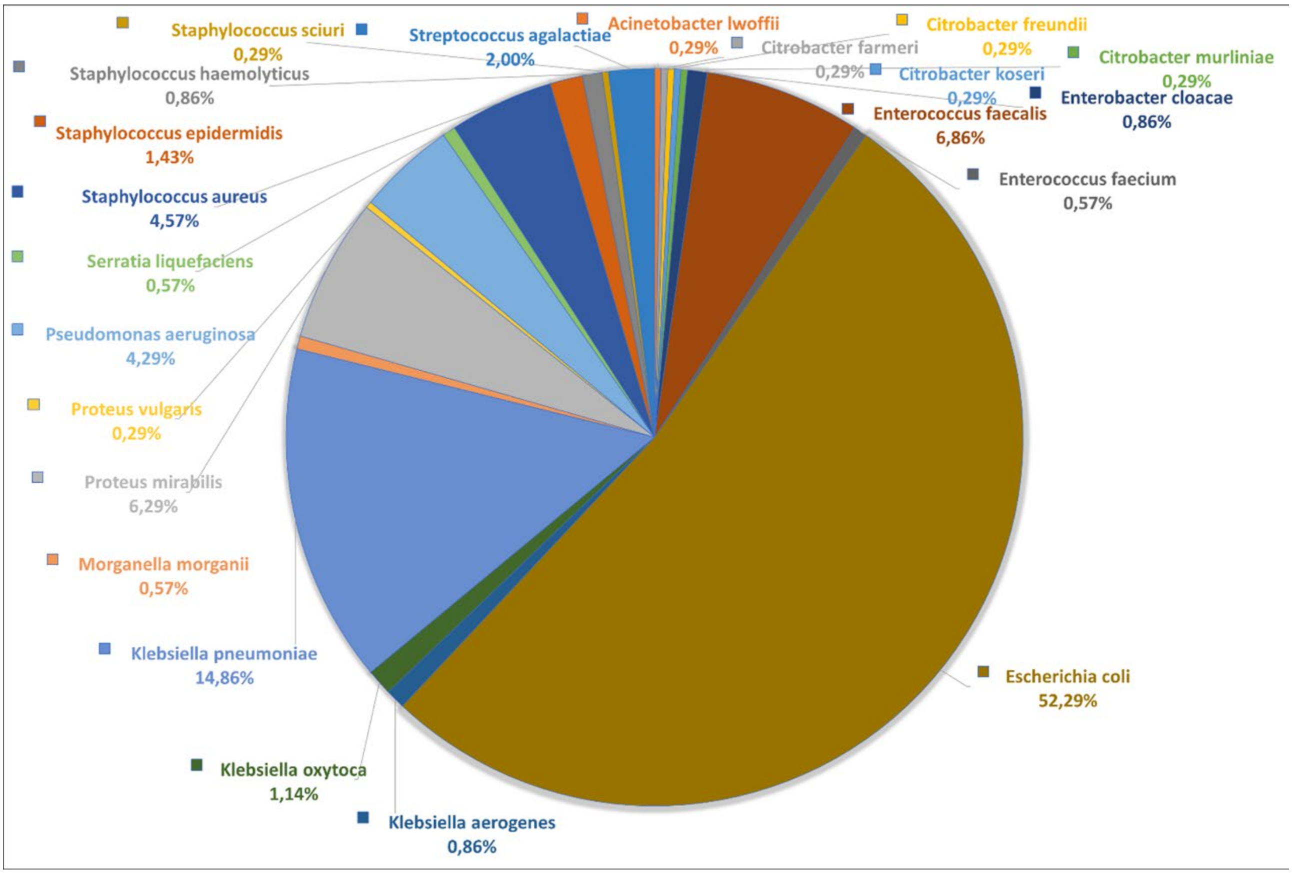

Figure S1: Representation of the percentages of identified bacterial strains in antibiogram samples.

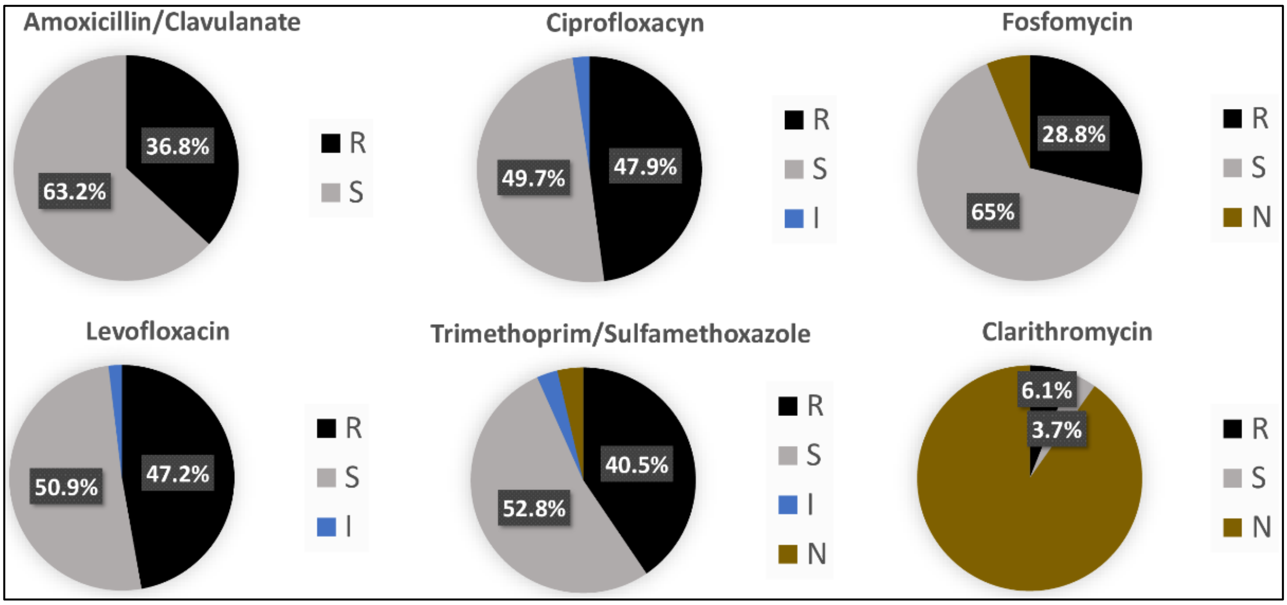

Figure S2: Percentage of resistance and sensitivity in male patients. R=resistance, S=sensitivity, I=Intermediate, N=Not available.

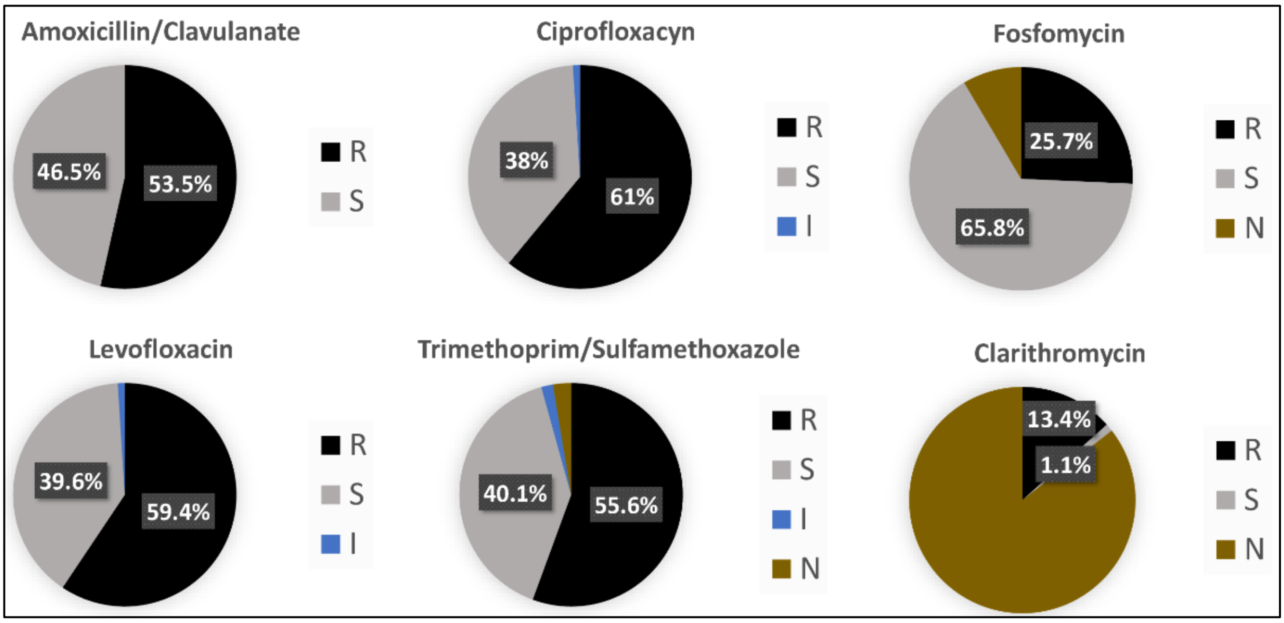

Figure S3: Percentage of resistance and sensitivity in female patients. R=resistance, S=sensitivity, I=Intermediate, N=Not available.

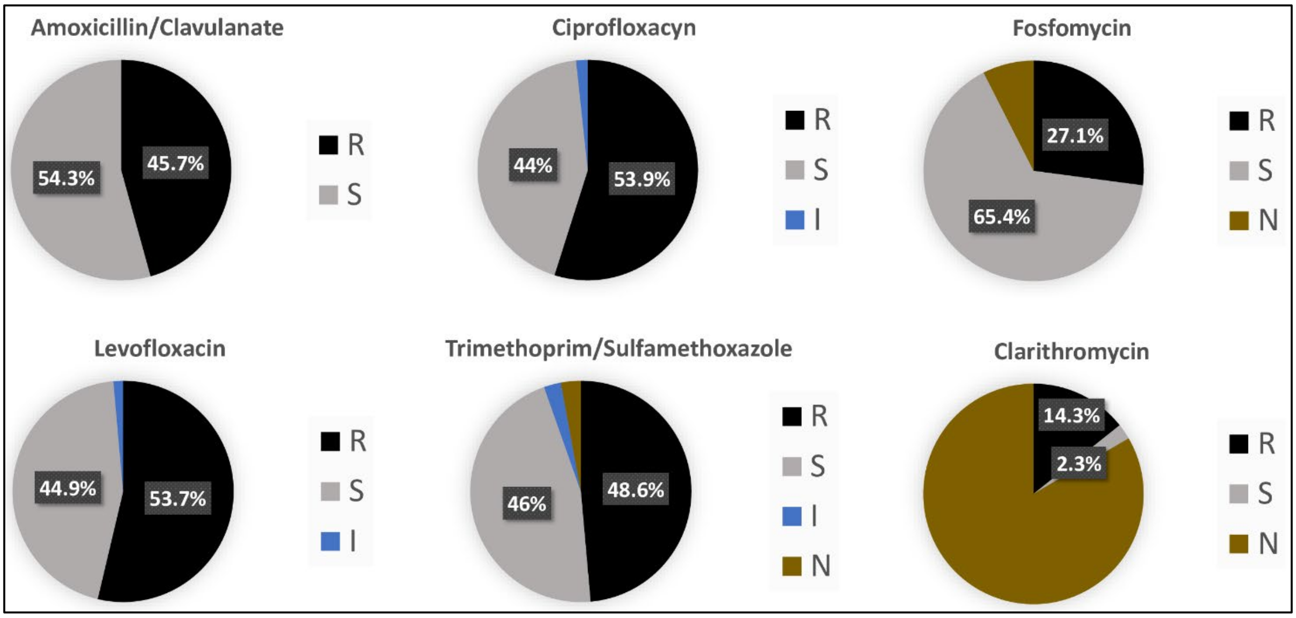

Figure S4: Percentage of resistance and sensitivity in total samples (males and females). R=resistance, S=sensitivity, I=Intermediate, N=Not available.

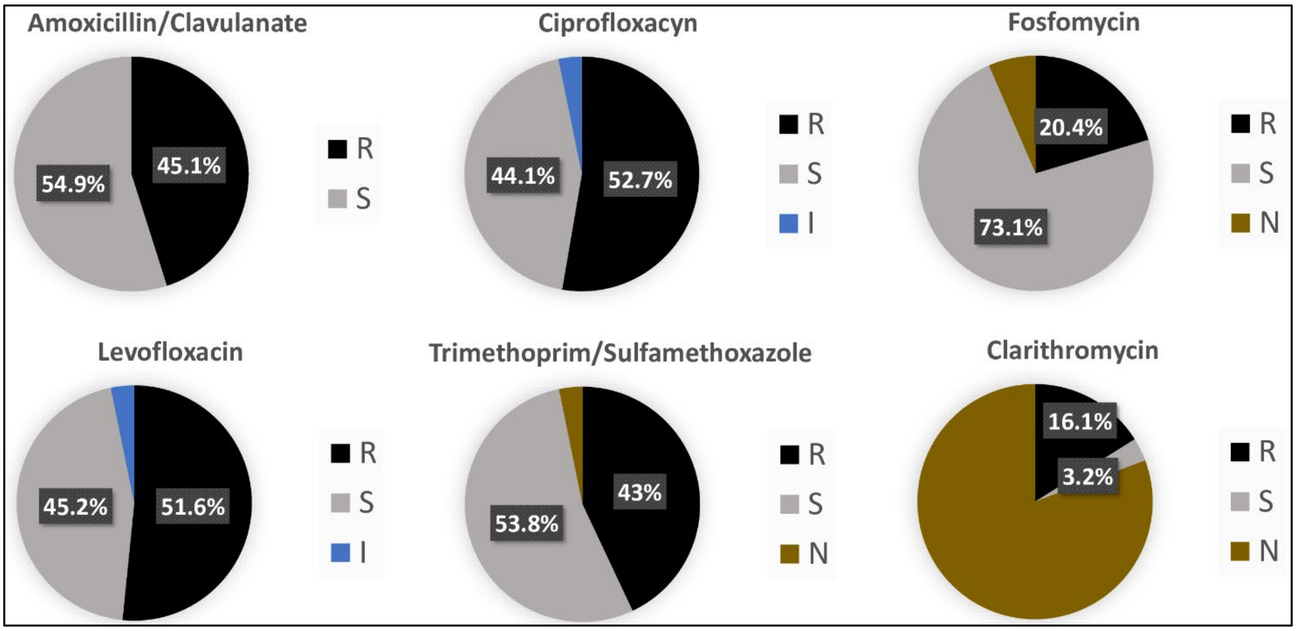

Figure S5: Percentage of resistance and sensitivity in patients aged ≤70 years old. R=resistance, S=sensitivity, I=Intermediate, N=Not available.

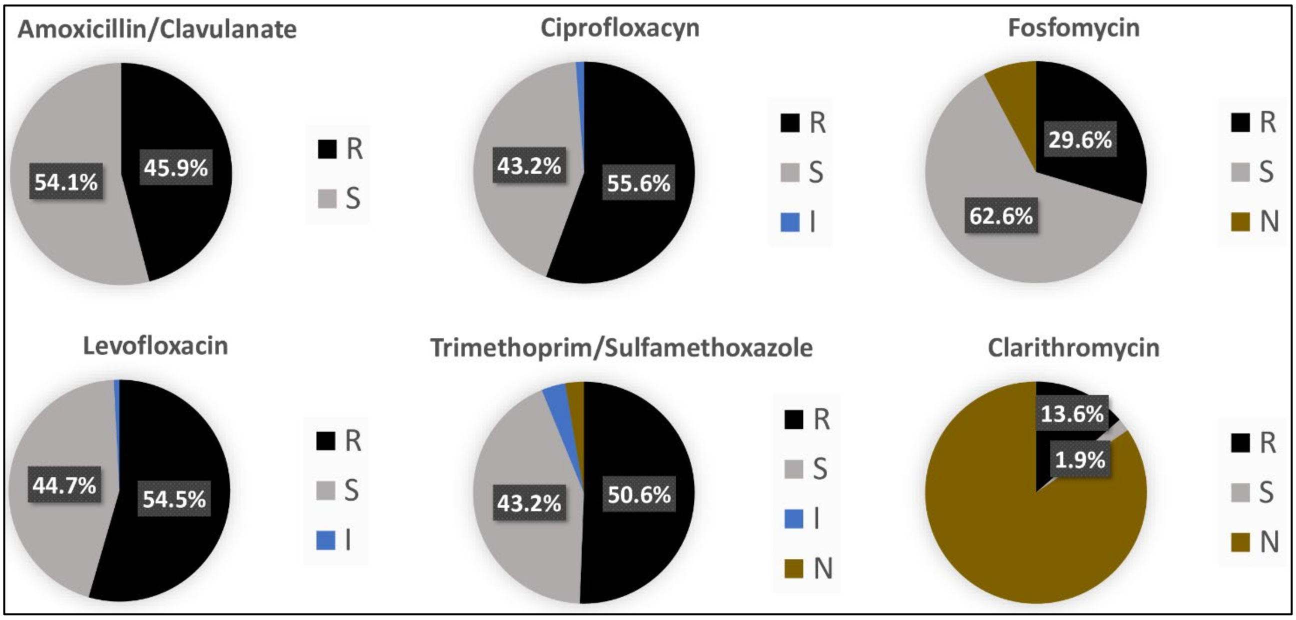

Figure S6: Percentage of resistance and sensitivity in patients aged >70 years old. R=resistance, S=sensitivity, I=Intermediate, N=Not available.

Table S1. Bacterial strains of the antibiogram samples.

Bacteria	(N)	(%)
Cinetobacter lwoffii	1	0.3
Citrobacter farmeri	1	0.3
Citrobacter freundii	1	0.3
Citrobacter koseri	1	0.3
Citrobacter murliniae	1	0.3
Enterobacter cloacae	3	0.9
Enterococcus faecalis	24	6.9
Enterococcus faecium	2	0.6
Escherichia coli	183	52.3
Klebsiella aerogenes	3	0.9
Klebsiella oxytoca	4	1.1
Klebsiella pneumoniae	52	14.9
Morganella morganii	2	0.6
Proteus mirabilis	22	6.3
Proteus vulgaris	1	0.3
Pseudomonas aeruginosa	15	4.3
Serratia liquefaciens	2	0.6
Staphylococcus aureus	16	4.6
Staphylococcus epidermidis	5	1.4
Staphylococcus haemolyticus	3	0.9
Staphylococcus sciuri	1	0.3
Streptococcus agalactiae	7	2.0

R=resistant, S=sensitive, N=number of samples, %=percentage of samples.

Table S2. Antibiotic sensitivity in male patients.

Antimicrobial	Interpretation	Male ≤ 70 (N)	Male ≤ 70 (%)	Male > 70 (N)	Male > 70 (%)	Total (N)	Total (%)	P-value
Amoxicillin/Clavulanate	R	12	20.0	48	80.0	60	36.8	0.624
	S	24	23.3	79	76.7	103	63.2	
Ciprofloxacin	R	20	25.6	58	74.4	78	47.9	0.278
	S	15	18.5	66	81.5	81	49.7	
Fosfomycin	R	9	19.1	38	80.9	47	28.8	0.464
	S	26	24.5	80	75.5	106	65.0	
Levofloxacin	R	19	24.7	58	75.3	77	47.2	0.409
	S	16	19.3	67	80.7	83	50.9	
Trimethoprim/Sulfamethoxazole	R	16	24.2	50	75.8	66	40.5	0.887
	S	20	23.3	66	76.7	86	52.8	
Clarithromycin	R	7	70.0	3	30.0	10	6.1	0.300
	S	3	50.0	3	50.0	6	3.7	

Table S3. Antibiotic sensitivity in female patients.

Antimicrobial	Interpretation	Female ≤ 70 (N)	Female ≤ 70 (%)	Female > 70 (N)	Female > 70 (%)	Total (N)	Total (%)	P-value
Amoxicillin/Clavulanate	R	30	30.0	70	70.0	100	53.5	0.878
	S	27	31.0	60	69.0	87	46.5	
Ciprofloxacin	R	29	25.4	85	74.6	114	61.0	0.105
	S	26	36.6	45	63.4	71	38.0	
Fosfomycin	R	10	20.8	38	79.2	48	25.7	0.089
	S	42	34.1	81	65.9	123	65.8	
Levofloxacin	R	29	26.1	82	73.9	111	59.4	0.189
	S	26	35.1	48	64.9	74	39.6	
Trimethoprim/Sulfamethoxazole	R	24	23.1	80	76.9	104	55.6	0.014
	S	30	40.0	45	60.0	75	40.1	*
Clarithromycin	R	8	32.0	17	68.0	25	13.4	0.183
	S	0	0.0	2	100.0	2	1.1	

R=resistant, S=sensitive, N=number of samples, %=percentage of samples. *p<0.05. Chi-square test.

Table S4. Antibiotic sensitivity and gender difference.

Antimicrobial	Interpretation	Male (N)	Male (%)	Female (N)	Female (%)	Total (N)	Total (%)	P-value
Amoxicillin/Clavulanate	R	60	37.5	100	62.5	160	45.7	0.002
	S	103	54.2	87	45.8	190	54.3	**
Ciprofloxacin	R	78	40.6	114	59.4	192	54.9	0.019
	S	81	53.3	71	46.7	152	43.4	*
Fosfomycin	R	47	49.5	48	50.5	95	27.1	0.601
	S	106	46.3	123	53.7	229	65.4	
Levofloxacin	R	77	41.0	111	59.0	188	53.7	0.027
	S	83	52.9	74	47.1	157	44.9	*
Trimethoprim/ Sulfamethoxazole	R	66	38.8	104	61.2	170	48.6	0.007
	S	86	53.4	75	46.6	161	46.0	**
Clarithromycin	R	25	50.0	25	50.0	50	14.3	0.188
	S	6	75.0	2	25.0	8	2.3	

R=resistant, S=sensitive, N=number of samples, %=percentage of samples, *p<0.05, **p<0.01. Chi-square test.

Table S5. Antibiotic sensitivity and gender difference in patients aged ≤70 years old.

Antimicrobial	Interpretation	Male ≤ 70 (N)	Male ≤ 70 (%)	Female ≤ 70 (N)	Female ≤ 70 (%)	Total (N)	Total (%)	P-value
Amoxicillin/Clavulanate	R	12	28.6	30	71.4	42	45.2	0.068
	S	24	47.1	27	52.9	51	54.8	
Ciprofloxacin	R	20	40.8	29	59.2	49	52.7	0.681
	S	15	36.6	26	63.4	41	44.1	
Fosfomycin	R	9	47.4	10	52.6	19	20.4	0.472
	S	26	38.2	42	61.8	68	73.1	
Levofloxacin	R	19	39.6	29	60.4	48	51.6	0.885
	S	16	38.1	26	61.9	42	45.2	
Trimethoprim/ Sulfamethoxazole	R	16	40.0	24	60.0	40	43.0	0.999
	S	20	40.0	30	60.0	50	53.8	
Clarithromycin	R	7	46.7	8	53.3	15	16.1	0.089

	S	3	100.0	0	0.0	3	3.2	

R=resistant, S=sensitive, N=number of samples, %=percentage of samples. Chi-square test.

Table S6. Antibiotic sensitivity and gender difference in patients aged >70 years.

Antimicrobial	Interpretation	Male > 70 (N)	Male > 70 (%)	Female > 70 (N)	Female > 70 (%)	Total (N)	Total (%)	P-value
Amoxicillin/Clavulanate	R	48	40.7	70	59.3	118	45.9	0.009
	S	79	56.8	60	43.2	139	54.1	**
Ciprofloxacin	R	58	40.6	85	59.4	143	55.6	0.002
	S	66	59.5	45	40.5	111	43.2	**
Fosfomycin	R	38	50.0	38	50.0	76	29.6	0.964
	S	80	49.7	81	50.3	161	62.6	
Levofloxacin	R	58	41.4	82	58.6	140	54.5	0.007
	S	67	58.3	48	41.7	115	44.7	**
Trimethoprim/ Sulfamethoxazole	R	50	38.5	80	61.5	130	50.6	0.001
	S	66	59.5	45	40.5	111	43.2	**
Clarithromycin	R	18	51.4	17	48.6	35	13.6	0.719
	S	3	60.0	2	40.0	5	1.9	

R=resistant, S=sensitive, N=number of samples, %=percentage of samples, *p<0.05, **p<0.01. Chi-square test.
